# Distal Locked versus Unlocked Intramedullary Nailing in Intertrochanteric Fracture; A Systematic Review and Meta-Analysis of Randomized and Non-Randomized Trials

**DOI:** 10.30476/BEAT.2020.46444

**Published:** 2020-04

**Authors:** Dushyant Chouhan, Sanjay Meena, Kulbhushan Kamboj, Mukesh Kumar Meena, Amit Narang, Siddhartha Sinha

**Affiliations:** 1 *Department of Orthopaedics, Lady Hardinge Medical college and associated hospitals, New Delhi, India*

**Keywords:** Intertrochanteric fractures, Intramedullary nail, Distal unlocking

## Abstract

**Objective:**

To compare the outcome of distal locked and unlocked intramedullary nailing in patients with intertrochanteric fractures through systematic review and meta-analysis of randomized and non-randomized clinical trials.

**Methods:**

Randomized or non-randomized controlled studies comparing the effects of unlocked and locked nails for treatment of intertrochanteric fractures were searched using the search strategy of Cochrane collaboration up to April 2019. Four eligible studies involving 691 patients were included. Their methodological quality was assessed, and data were extracted independently for meta-analysis.

**Results:**

The results showed that the unlocked group has significantly less operative time (MD: -8.08; 95%CI -11.36 to -4.79; P< 0.00001), fluoroscopy time (MD: -7.09, 95%CI -7.09 to -4.79; *p*<0.00001), length of incision (MD: -2.50, 95%CI 2.85 to -2.14; *p*< 0.00001) than the locked group. The complication rate was significantly higher in the locking group (OR: 0.55, 95%CI 0.26 to 1.15; *p*=0.03). No significant differences were found in the Harris hip score between the two groups (MD: 0.68, 95% CI -0.83 to 2.19, *p*<0.08).

**Conclusion:**

The present meta-analysis suggests that intramedullary nailing without distal locking is reliable and acceptable option for treating intertrochanteric fracture. The advantages are reduced operative time, decreased fluoroscopy time, smaller size of incision and decreased complication rate. However, owing to the low-quality evidence currently available, additional high quality Randomized controlled trials are needed to confirm these findings.

## Introduction

Intertrochanteric fracture is one of the most common fracture around hip [1]. It frequently occurs in the geriatric population and is often associated with generalized physical deterioration. With increasing life expectancy, the number of aged individuals are increasing and according to one estimate, the hip fractures will grow from 1.66 million in 1990 to 6.26 million by 2050 [2]. 

Either intramedullary or extramedullary device is used for surgical treatment of intertrochanteric fractures. The most commonly used extramedullary implant used is dynamic hip screw. Intramedullary devices such as proximal femoral nail and gamma nail have a biomechanical advantage over extramedullary implants because of their short lever arm and reduced deforming forces across the nail. But distal locking screws used in intramedullary nail can act as a stress riser that can cause complications such as implant breakage and may also lead to fascia Lata irritation [3-6]. Although the effects of distal unlocking in intramedullary nailing for intertrochanteric fractures have been reported, the results and conclusion are not consistent [7-11]. The controversy regarding whether distal locking screws are necessary is still not settled. Therefore, we conducted this meta-analysis to investigate whether there is a significant difference between unlocked and locked intramedullary nail in treatment of intertrochanteric fracture.

## Materials and Methods

This meta-analysis was reported according to the preferred reporting items for systematic reviews and meta-analyses (PRISMA) guidelines. All analyses were based on previous published studies, thus no ethical approval and patient consent are required

*Search strategy*

We searched for studies comparing the effects of unlocked and locked nail used for intertrochanteric fracture according to the search strategy of the Cochrane collaboration. It included searching of the Cochrane Musculoskeletal Injuries Group Trials Register, computer searching of MEDLINE, EMBASE, and Current Contents, and hand searching of orthopaedic journals. The following key words are used in combination with Boolean operators AND or OR: “intertrochanteric fracture “, intramedullary nailing”, “nail” and “distal unlocking”. References in the included articles were also scanned for potentially relevant studies. No restrictions were placed on the publication language. Two reviewers (DK and SM) independently assessed the titles and abstracts of all the reports identified by the electronic and manual searches. Subsequently, the full-text of potential articles which meet the inclusion criteria was screened, and a final decision was made. Disagreements were resolved by consulting a third reviewer (KK).

*Inclusion criteria and study selection*

All databases were searched from the earliest records to April 2019. The inclusion and exclusion criteria used in selecting eligible studies were: (1) target population – individuals with intertrochanteric fractures, excluding sub trochanteric and pathological fractures; (2) intervention – unlocked intramedullary nail fixation compared with locked intramedullary nail fixation; (3) methodological criteria– randomized or non-randomized controlled study; (4) duplicate or multiple publications of the same study were not included.

*Data extraction*

Data were collected by 2 independent researchers (DK, SM) who screened titles, abstracts, and keywords both electronically and by hand; differences were resolved by third researcher (KK). Full texts of citations that could possibly be included in the present meta-analysis were retrieved for further analysis. The primary outcome was operative time and post-operative complications. The secondary outcome was blood loss, fluoroscopy time, and length of incision and Harris hip score.

*Quality Assessment*

We used modified jaded scale for quality assessment of studies [12]. This is an 8-item scale that was designed to evaluate randomization, blinding, inclusion and exclusion criteria, withdrawals and dropouts, adverse effects, and statistical analysis. Scores range from 0 (lowest quality) to 8 (highest quality), and 4-8 represent good or high quality, whereas 0-3 symbolize poor or low quality.

*Statistical Analysis*

We did not undertake a subgroup analysis for different fracture types because not all the studies included described the fracture types. In each eligible study the relative risk (RR) was calculated for dichotomous outcomes and the weighted mean difference for continuous outcomes using the software Review Manager 5.0, with a 95% confidence interval (CI) adopted in both. Heterogeneity was tested using both the chi-square test and the I-square test. A significance level of less than 0.10 for the chi-square test was interpreted as evidence of heterogeneity. The I-square was used to estimate total variation across studies. When there was no statistical evidence of heterogeneity, a fixed-effect model was adopted; otherwise, a random-effect model was chosen. We did not include the possibility of publishing bias due to the small number of studies included.

## Results

**Fig. 1:**
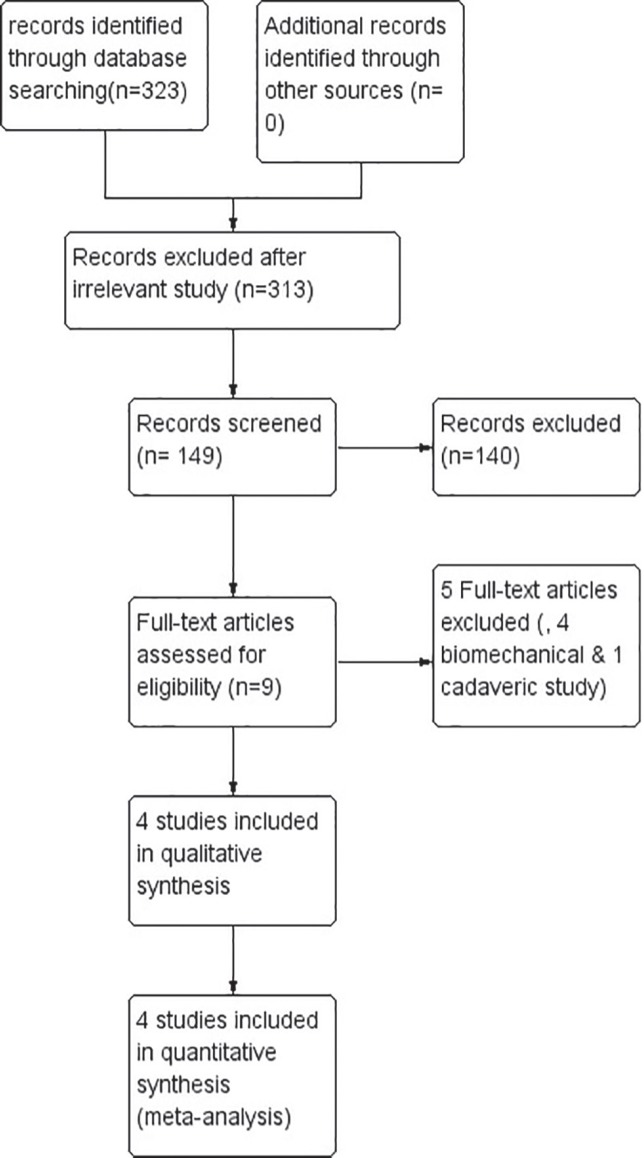
Search results and the selection procedure

A total of four articles comparing the effects of unlocked and locked nail used for intertrochanteric fracture were retrieved. The search process is presented in Figure 1 and the general characteristics of studies analysed are given in Table 1. The quality assessment using modified jaded score is presented in Table 2.

*Operative time*

**Fig. 2:**
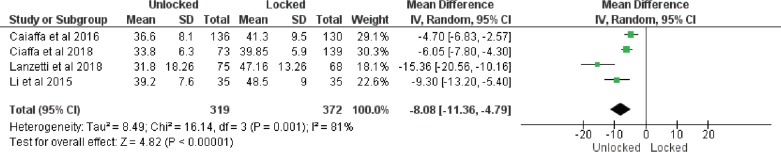
Forest plot of operative time

Four studies provided data on operative time. The random effects model was used because of the statistical heterogeneity (I^2^=81%). The meta-analysis indicated that the operative time in unlocked group was significantly shorter than locked group (MD: -8.08, 95% CI: -11.36 to -4.79, *p*<0.00001) Figure 2.

*Fluoroscopy time*

**Fig. 3:**
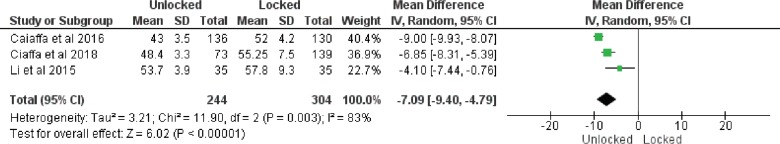
Forest Plot of fluoroscopy time

Three studies provided data on Fluoroscopy time. The random effects model was used because of the statistical heterogeneity (I^2^=83%). The meta-analysis indicated that the fluoroscopy time in unlocked group was significantly shorter than locked group (MD: -7.09, 95% CI: -7.09 to -4.79, *p*<0.00001) (Figure 3).

**Fig. 4:**
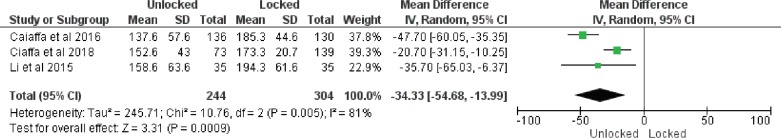
Forest plot of blood loss

Three studies provided data on intraoperative blood loss. The random effect model was used because of the statistical heterogeneity (I^2^=81%). The meta-analysis indicated that the intraoperative blood loss was significantly less in unlocked group. (MD: -34.33, 95% CI: 54.68 to -13.09, *p*=0.0009) (Figure 4).

*Length of incision*

**Fig. 5:**
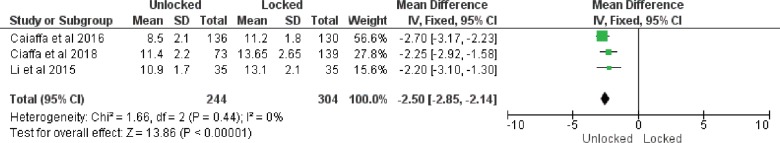
Forest plot of length of incision

Three studies provided data on length of incision. The fixed effect model was used because of the statistical heterogeneity (I^2^=0%). The meta-analysis indicated that the length of incision in the unlocked group was significantly shorter than locked group (MD: -2.50, 95% CI: 2.85 to - 2.14, *p*<0.00001) (Figure 5).

*Harris hip score*

**Fig. 6:**
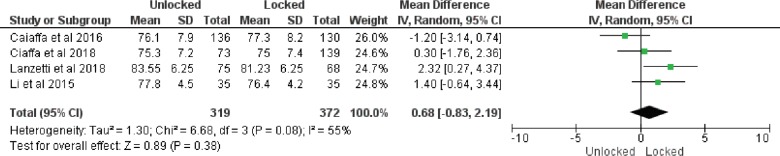
Forest plot of Harris hip score

Four studies provided data on Harris Hip score. The random effect model was used because of the statistical heterogeneity (I^2^=55%). The meta-analysis indicated that the there was no significant difference in terms of Harris hip score between the two groups (MD: 0.68, 95% CI: -0.83 to 2.19, *p*=0.08) (Figure 6).

*Complication rates*

**Fig. 7:**
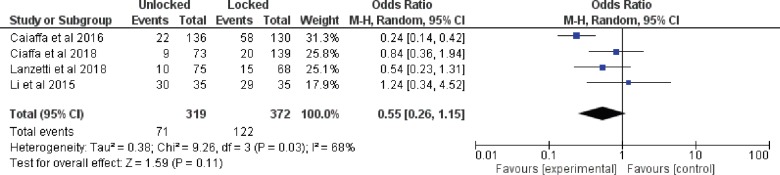
Forest plot of complications

Four studies provided data on complication rate. The random effect model was used because of the statistical heterogeneity (I^2^=68%). The meta-analysis indicated that unlocked group had significantly less complications than locked group (OR: 0.55, 95% CI: 0.26 to 1.15, *p*=0.03) (Figure 7).

**Table 1:** Characteristics of the included studies

**Studies **	**Age** **(Unl/Loc)**	**No of patients** **(Unl** ^c^ **/Loc)**	**Gender** **(M/F)**	**Study type**	**Fracture type** **A1/A2**
**Ciaffa et al ,2018**	75.6 ±3.4/76.8 ±2.25	73/139	138/74	RCT^a^	47/94
**Lanzetti et al 2018**	85.48 ± 7.84/ 84.5 ±8.76	75/68	24/119	RCT^a^	73/70
**Ciaffa et al 2016**	77.9±7.2/ 78.4 ±7.1	136/130	93/174	RCT^a^	85/181
**Li et al 2015**	78.3 ±7/78.1 ±6.9	35/35	21/49	PRT^b^	17/52

^a^RCT: Randomized controlled trial; ^b^PRT: Prospective randomized study; ^c^Unl: Unlocked; ^d^Loc: Locked

**Table 2:** Quality evaluation according to the modified JADAD scale

**Item Assessed**	**Caiaffa et al. ,2016 [** 9 **]**	**Li et al. ,2015 [** 8 **]**	**Caiaffa et al. ,2018 [** 11 **]**	**Lanzetti et al. ,2018 [** 10 **]**
Was the study described as randomised?	YES	YES	YES	NO
Was the method of randomization appropriate?	YES	YES	YES	Not described
Was the study described as blinded?	NO	NO	NO	NO
Was the method of blinding appropriate?	Not described	Not described	Not described	Not described
Was there a description of withdrawals and dropouts?	YES	YES	YES	YES
Was there a clear description of inclusion and exclusion criteria?	YES	YES	YES	YES
Was the method used to assess adverse effect described?	YES	YES	YES	YES
Was the method of statistical analysis described?	YES	YES	YES	YES
TOTAL SCORES	6	6	6	4

**Legends for figures**:

## Discussion

The increasing incidence of intertrochanteric fracture is a global health issue because of the high morbidity and mortality associated with this fracture. There is increasing trend among orthopaedic surgeons to use minimally invasive surgical techniques which allow them to decrease soft tissue damage and reduce operative time, blood loss and complications. This have led to the increasing popularity of intramedullary nail in intertrochanteric fractures [13]. The distal locking screw used in intramedullary nailing for intertrochanteric fractures may lead to increase in mechanical stress which may further lead to hypertrophy of surrounding cortical bone, pain in fascia lata and fractures around screw [14,15].

Intramedullary nails are load bearing implant and work as internal splints. The load bearing ability of any intramedullary nail depends on the fracture characteristics and the achieved reduction [16]. In stable fractures a significant cortical contact is attained and a large portion of compressive loads will be transmitted through the cortices; however, in unstable fractures where cortical contact is minimal, compressive loads are transmitted distally through the nail to the distal interlocking screws [17]. Moreover, the lag screws pass at the same time through the lateral cortex of the distal fragment and thus stabilise both fragments and prevent their mutual rotation and longitudinal compression along the nail, making distal locking not necessary [18]. However distal locking screw should be used in comminuted, reverse obliquity and high obliquity fractures characterised by femoral shortening and rotational instability. Distal locking is also necessary in patients with severe osteoporosis. Lacroix *et al*. in his cadaveric study showed that an additional hole in the distal part of the nail could decrease the mean failure load in torsion by 36% as a stress raiser [19].

This analysis showed that the unlocked group have significantly less operative time, decreased blood loss, decreased fluoroscopy time and smaller length of incision. Decrease in operative time was expected as distal locking step was not done in unlocked group. This is also the cause for smaller length of incision as separate incision for distal locking is not required. Fluoroscopy exposure is decreased as distal locking is usually done under fluoroscopic guidance. However, there was no significant difference in the Harris hip score. Further important observation is the decreased complication rate in the unlocked group. The most common complication in the locked group was thigh pain. This may be due to iliotibial tract irritation or cortical hypertrophy. Also, there is increasing tendency of screw cut out in locked group. One of the most feared complication following intramedullary nailing in intertrochanteric fractures is the diaphyseal fracture around the distal part of the nail. There was 2.3% (n=7) incidence of sub trochanteric fracture in locked as compared to 1%(n=2) in unlocked group. Although the difference was not significant but there is a risk of subsequent diaphyseal fractures around the distal part of the nail. This may me because of the bone weakening caused due to excessive tightening of the distal screw and the excessive reaming of the medullary canal. Most of these fractures occur within three months from surgery [20, 21].

Our results led us to believe that using intramedullary nails without distal locking is a reliable and acceptable option in operative treatment of stable intertrochanteric fractures. Apart from reducing complications, this practice can also provide other advantages such as decreased operative time, blood loss and length of incision. Nevertheless, this current meta-analysis showed that, overall, there was no statistically significant difference between the surgical groups in the complication rate.

There are several limitations in our meta-analysis. Firstly, the number of studies included, and the sample size of patients were quite limited. In addition, the 4 studies were of relatively poor quality, which might weaken the strength of the findings. Secondly, we did not undertake a subgroup analysis of different fracture types because not all the studies included described the fracture types. Furthermore, not all the studies included had long enough follow-up periods, which also reduces the power of our research.

Although the evidence quality was ‘very low’ in this meta-analysis because of clinical heterogeneity and limited information, the data tend to suggest that intramedullary nails without distal locking may be superior to distal locked nail for the Intertrochanteric femur fractures. Further research is required and future without distal locking studies should include analysis of assessments at 12 to 24 months and follow-up after removal of the implant.

The present meta-analysis suggests that intramedullary nailing without distal locking is reliable and acceptable option for treating intertrochanteric fracture. The advantages are decrease operative time, fluoroscopy time, size of incision and decreased complication rate. However, owing to the low-quality evidence currently available, additional high quality RCTS are needed to confirm these findings.

## Conflict of interest:

The authors disclose that we do not have any conflict of interest.

## References

[1] Cummings SR, Rubin SM, Black D (1990). The future of hip fractures in the United States Numbers costs and potential effects of postmenopausal estrogen. ClinOrthop Relat Res.

[2] Kannus P, Parkkari J, Sievänen H, Heinonen A, Vuori I, Järvinen M (1996). Epidemiology of hip fractures. Bone.

[3] Heinz T, Vécsei V (1994). Complications and errors in use of the gamma nail Causes and prevention. Chirurg.

[4] Hesse B, Gächter A (2004). Complications following the treatment of trochanteric fractures with the gamma nail. Arch Orthop Trauma Surg.

[5] Lacroix H, Arwert H, Snijders CJ, Fontijne WP (1995). Prevention of fracture at the distal locking site of the gamma nail A biomechanical study. J Bone Joint Surg Br.

[6] Radford PJ, Needoff M, Webb JK (1993). A prospective randomised comparison of the dynamic hip screw and the gamma locking nail. J Bone Joint Surg Br.

[7] Skála-Rosenbaum J, Bartonícek J, Bartoska R (2010). Is distal locking with IMHN necessary in every pertrochanteric fracture?. Int Orthop.

[8] Li X, Zhang L, Hou Z, Meng Z, Chen W, Wang P (2015). Distal locked and unlocked nailing for perthrochanteric fractures--a prospective comparative randomized study. Int Orthop.

[9] Caiaffa V, Vicenti G, Mori C, Panella A, Conserva V, Corina G (2016). Is distal locking with short intramedullary nails necessary in stable pertrochanteric fractures? A prospective, multicentre, randomised study. Injury.

[10] Lanzetti RM, Caraffa A, Lupariello D, Ceccarini P, Gambaracci G, Meccariello L (2018). Comparison between locked and unlocked intramedullary nails in intertrochanteric fractures. Eur J Orthop Surg Traumatol.

[11] Ciaffa V, Vicenti G, Mori CM, Panella A, Conserva V, Corina G (2018). Unlocked versus dynamic and static distal locked femoral nails in stable and unstable intertrochanteric fractures A prospective study. Injury.

[12] Oremus M, Wolfson C, Perrault A, Demers L, Momoli F, Moride Y (2001). Interrater reliability of the modified Jadad quality scale for systematic reviews of Alzheimer's disease drug trials. Dement Geriatr Cogn Disord.

[13] Schipper IB, Steyerberg EW, Castelein RM, van der Heijden FH, den Hoed PT, Kerver AJ (2004). Treatment of unstable trochanteric fractures Randomised comparison of the gamma nail and the proximal femoral nail. J Bone Joint SurgBr.

[14] Robinson CM, Adams CI, Craig M, Doward W, Clarke MC, Auld J (2002). Implant-related fractures of the femur following hip fracture surgery. J Bone Joint SurgAm.

[15] Barry TP (1984). Radiation exposure to an orthopedic surgeon. Clinical orthopaedics andrelated research.

[16] Kane P, Vopat B, Paller D, Koruprolu S, Daniels AH, Born C (2014). A biomechanical comparison of locked and unlocked long cephalomedullary nails in a stable intertrochanteric fracture model. J Orthop Trauma.

[17] Bong MR, Kummer FJ, Koval KJ, Egol KA (2007). Intramedullary nailing of the lower extremity: biomechanics and biology. J Am Acad Orthop Surg.

[18] Skála-Rosenbaum J, Bartoníček J, Bartoška R (2010). Is distal locking with IMHN necessary in every pertrochanteric fracture?. International orthopaedics.

[19] Lacroix H, Arwert H, Snijders CJ, Fontijne WP (1995). Prevention of fracture at the distal locking site of the gamma nail A biomechanical study. J Bone Joint Surg Br.

[20] Albareda J, Laderiga A, Palanca D, Paniagua L, Seral F (1996). Complications and technical problems with the gamma nail. Int Orthop.

[21] Saarenpää I, Heikkinen T, Jalovaara P (2007). Treatment of subtrochanteric fractures A comparison of the Gamma nail and the dynamic hip screw: short-term outcome in 58 patients. Int Orthop.

